# Trichobezoar from bristles brush and Carpet yarn requiring emergency laparotomy. Case report

**DOI:** 10.1016/j.amsu.2021.102192

**Published:** 2021-02-24

**Authors:** Kusay Ayoub, Abdelaziz Alibraheem, Esraa Masri, Amira Kazan, Samer Rajab Basha, Mahmoud Hamoud, Nihad Mahli

**Affiliations:** a PHD, Department of Surgery, Faculty of Medicine, University of Aleppo, Syria; b Faculty of Medicine, University of Aleppo, Aleppo, Syria; c MD, Department of Surgery, Faculty of Medicine, University of Aleppo, Syria; d Professor of surgery, Department of Surgery, Faculty of Medicine, University of Aleppo, Syria

**Keywords:** Abdominal mass, Trichophagia, Trichobezoar, Trichotillomania

## Abstract

**Introduction and importance:**

When hair accumulates inside the stomach, it causes what is called a Trichobezoar, which leads to a stomach blockage, this condition is rare and more common in women and in patients with psychiatric disorders.

**Case presentation:**

The authors report an unusual case of a 16-year-old girl who has trichobezoar not only by ingestion of hair, it is also by bristle clothes brush and Carpet yarn. she presented with acute abdominal pain and gastrointestinal symptoms-like watery diarrhea, vomiting, hypercoria and weight loss attributed to Anorexia. With an upper gastroscopy, the condition was diagnosed as a huge Trichobezoar that occupied the stomach. The patient was managed by surgical removal of the intra gastric mass.

**Clinical discussion:**

Affected patients infrequently remain asymptomatic for several years. Symptoms begin while the bezoar increases in size to the point of obstruction, these symptoms are nonspecific like vomiting, nausea, anorexia, asymptomatic abdominal mass and digestive bleeding.

**Conclusion:**

Trichobezoar considers as a differential diagnosis for any patient with psychological disorders, like trichotillomania and trichophagia and has gastrointestinal symptoms.

## Highlights

• Trichobezoar considers as a differential diagnosis for any patient with psychological disorders, like trichotillomania and trichophagia and has gastrointestinal symptoms.
• Trichobezoar is caused by chronic ingestion of hair; Small trichobezoars may be extracted by endoscopic fragmentation, huge trichobezoar, on the other hand need surgical removal.
• Early diagnosis and an appropriate therapy can reduce morbidity and mortality.
• Psychological counselling plays a pivotal role in order to prevent bezoar recurrence

## 1 Introduction

Our paper has been reported according to SCARE criteria [1].

Bezoar is an abnormal condition, in which non-digestible substances accumulate inside the gastrointestinal tract causing its blockage.

In the absence of adequate treatment, the associated mortality rate is up to 30%, principally because of gastrointestinal bleeding, destruction, or perforation [2].

There are various types of bezoars, one of which is Trichobezoar, in which there is a gathering of hair inside the gastrointestinal tract. This condition is more common in animals than in humans, and this type of bezoar is the most common in humans [3], Trichobezoars are associated with trichophagia as a result of pica – an eating disorder manifested by an appetite for nonnutritive substances and often associated with mental alteration – and coexistent psychiatric disturbances [4].

The word “Trichobezoar” is a combination of “trich” meaning hair in Greek and “bezoar” meaning poison antidote in Arabic or Persian [5].

Here we present a case of trichobezoar in a 16-year-old girl who used to eat bristle clothes brush and her hair for 12 years.

## 2 Case report

A 16 – year – old female was referred to the surgical clinic with acute worsening Epigastric pain with refers to her shoulder. The pain started 2 years ago, associated with nausea, Intermittent vomiting, hyperthermia, hypercoria, indigestion along with Constipation.

Her parents described that the patient had habits of hair, bristles brush and Carpet Yarn pulling and chewing for 2 years.

The patient had no past medical history or drug history. The patient had stable vital signs. The Physical examination revealed a hard mass in the Epigastric region and signs of an acute abdomen with rebound tenderness and pain on the removal of pressure. There were hairless regions on her scalp in the frontal and parietal areas, bilaterally.

**Fig. 1:**
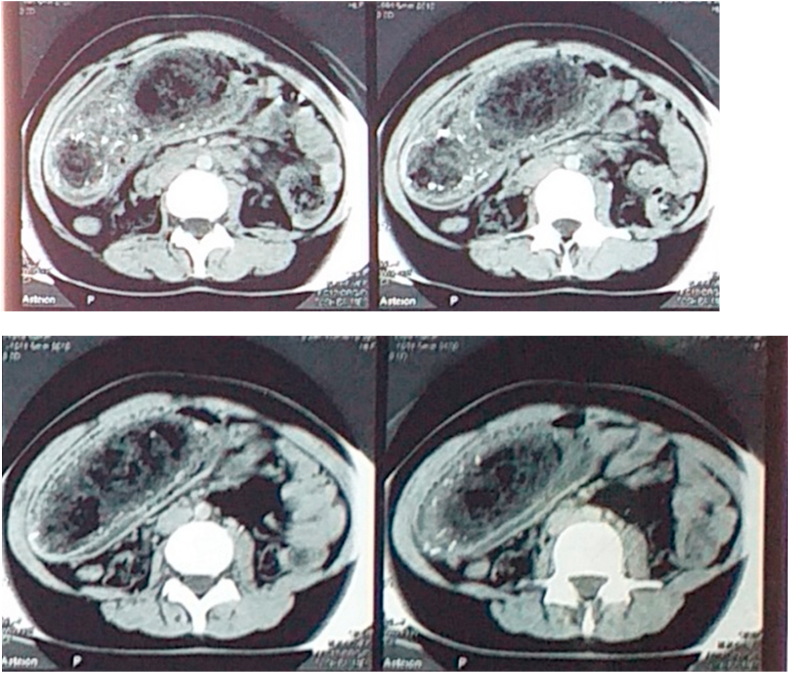
The stomach was distended and filled with a large solid mass.

**Fig. 2:**
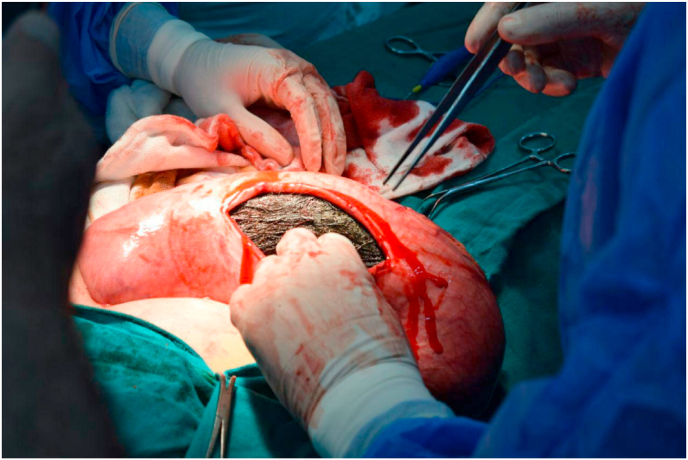
A giant trichobezoar with the shape of the stomach.

A Computed Tomography Scan CT (Fig. 1) was proceeded and has shown that the stomach was distended and filled with a large solid mass.

upper gastrointestinal endoscopy disclosed a large mass of trichobezoar occupies the stomach which could not be extracted or exceeded. Therefore, the patient underwent emergency laparotomy; an anterior gastrotomy was done (Fig. 2). There was a giant trichobezoar with the shape of the stomach. We closed the incision in two layers using 2-0 Vicryl suture and the abdomen was closed without drainage.

The patient was put under observation for 5 days in hospital then she was discharged with a good general condition.

## 3 Discussion

Bezoars are concretions of foreign substances in the gastrointestinal tract, mainly the stomach. Bezoars composed of hair or hair-like fibers are called 'trichobezoars'. Trichobezoars tend to appear in the second decade of life [4]. Often in females with psychiatric disorders including trichotillomania (pulling out their hair) and trichophagia (eating hair) [6].

Trichobezoars form when Ingested hair strands are accumulated in the gastric folds, escaping peristaltic propulsion because of their slippery surface, prevents enough friction which is required to push them out of the stomach. The Ingested hair becomes even more matted together and takes the shape of the stomach, usually as a single solid mass [7].

Affected patients infrequently remain asymptomatic for several years. Symptoms begin while the bezoar increases in size to the point of obstruction. the patient with a gastric trichobezoar usually presents with nonspecific symptoms, including abdominal pain (70%), nausea and vomiting (64%), digestive bleeding (61%), epigastric discomfort, early satiety, indigestion, weight loss (38%), diarrhea or constipation (32%) [4].

Complications by a huge eroding or obstructing bezoar additionally involve obstructive jaundice, severe anemia either due to malabsorption or gastrointestinal bleeding, acute pancreatitis, and gastric emphysema and this complication are infrequent and raise mortality rate to 30%.

Trichobezoar diagnosis is made by endoscopic examination and radiography imaging. Upper gastrointestinal (GI) endoscopy can provide information about the structure of the mass. The Computed tomography (CT) investigation can Reveal the existence, localization, and distribution of the bezoars [8].

Different therapeutic modalities have been suggested to treat trichobezoar like endoscopy, Surgery, and pharmacological approaches. Surgery by gastrotomy or enterotomy is still the mainstay for gastric trichobezoar removal especially those that extend into the intestine. Because of the enormous size of the mass, laparotomy was chosen as the surgical method in order to remove the whole trichobezoar mass successfully.

## 4 Conclusion

Trichobezoar is caused by chronic ingestion of hair; Small trichobezoars may be extracted by endoscopic fragmentation, huge trichobezoar, on the other hand need surgical removal. Early diagnosis and an appropriate therapy can reduce morbidity and mortality.

Psychological counselling plays a pivotal role in order to prevent bezoar recurrence.

## Declaration of competing interest

Authors declare that there is no conflict of interest.

## Registration of research studies

1. Name of the registry:
2. Unique Identifying number or registration ID:
3. Hyperlink to your specific registration (must be publicly accessible and will be checked):
