# Supplementary material for: Trichobezoar from bristles brush and Carpet yarn requiring emergency laparotomy. Case report
Source: Ann Med Surg (Lond). 2021 Feb 24;63:102192. doi: 10.1016/j.amsu.2021.102192 (PMC7930585; doi:10.1016/j.amsu.2021.102192)
Supplement: Multimedia component 1 [file mmc1.docx]

| **SCARE Checklist** | | | |
| --- | --- | --- | --- |
| **Topic** | **Item** | **Checklist item description** | **Page Number** |
| **Title** | **1** | Trichobezoar from bristles brush and Carpet Yarn Requiring Emergency Laparotomy. A Case Report | 1 |
| **Key Words** | **2** | **Abdominal Mass; trichophagia; Trichobezoar; Trichotillomania** | 1 |
| **Abstract** | **3a** | Trichobezoar considers as a differential diagnosis for any patient with psychological disorders, like trichotillomania and trichophagia and has gastrointestinal symptoms and may be the reason not only hair  It maybe bristles brush like this case. | 1 |
|  | **3b** | The authors report an unusual case of a 16-year-old girl who has trichobezoar not only by ingestion of hair, it is also by bristle clothes brush. she presented with acute abdominal pain and gastrointestinal symptoms |  |
|  | **3c** | With an upper gastroscopy, the condition was diagnosed as a huge Trichobezoar that occupied the stomach. The patient was managed by surgical removal of the intra gastric mass, and the patient is now in good condition. |  |
|  | **3d** | Trichobezoar considers as a differential diagnosis for any patient with psychological disorders, like trichotillomania and trichophagia and has gastrointestinal symptoms. |  |
| **Introduction** | **4** | Bezoar is an abnormal condition, in which non-digestible substances accumulate inside the gastrointestinal tract causing its blockage.  In the absence of adequate treatment, the associated mortality rate is up to 30%, principally because of gastrointestinal bleeding, destruction, or perforation | 2 |
| **Patient Information** | **5a** | A 16- year female | **2** |
|  | **5b** | referred to the surgical clinic with acute worsening Epigastric pain with refers to her shoulder.  The pain started 2 years ago, associated with nausea, Intermittent vomiting, hyperthermia, hypercoria, indigestion along with Constipation. |  |
|  | **5c** | The physical examination revealed a malnourished girl; vital signs were normal |  |
|  | **5d** | Drug history only the patient took pain relievers for several months due to her abdominal pain., family history including any relevant genetic information was clear, and psychosocial history including smoking status (no) and where relevant accommodation type (city) family history including relevant genetic information was clear. |  |
| **Clinical Findings** | **6** | The physical examination revealed a malnourished girl; vital signs were normal  The Physical examination revealed a hard mass in the Epigastric region and signs of an acute abdomen with rebound tenderness and pain on the removal of pressure.  There were hairless regions on her scalp in the frontal and parietal areas, bilaterally. | 2 |
| **Timeline** | **7** | The pain started 2 years ago, associated with nausea, Intermittent vomiting, hyperthermia, hypercoria, indigestion along with Constipation, and about 6 kg of weight loss within the last month. | 2 |
| **Diagnostic Assessment** | **8a** | Diagnostic methods (physical exam with Abdominal examination, radiological imaging (CT) Upper gastrointestinal endoscopy). | 2 |
|  | **8b** | Diagnostic challenges: no diagnostic challeges |  |
|  | **8c** | diagnoses considered (Trichobezoar) |  |
|  | **8d** | A computed tomography scan CT (figure 1) was proceeded and has shown that the stomach was distended and filled with a large solid mass. |  |
| **Therapeutic Intervention** | **9a** | The patient took pain relievers for several months due to her abdominal pain. | 2-3 |
|  | **9b** | Therefore, the patient underwent emergency laparotomy; an anterior gastrotomy was done  We closed the incision in two layers using 2-0 Vicryl suture and the abdomen was closed without drainage |  |
|  | **9c** | Peri-intervention, the patient underwent emergency laparotomy; an anterior gastrotomy was done (figure1). There was a giant trichobezoar with a long tail of hair extending within the pylorus into the proximal jejunum; the mass takes the shape of the stomach and duodenum. Due to this feature, “Rapunzel Syndrome” was the clear diagnosis. (figure2)  We closed the incision in two layers using 2-0 Vicryl suture and the abdomen was closed without drainage |  |
|  | **9d** | Kusay Ayoub PHD, DIU, CES ,MD Instructor General Surgery , Faculty of medicine, University of Aleppo, Syria, |  |
|  | **9e** | Any changes in the interventions with rationale. Include intra-operative photographs and/or video or relevant histopathology in this section. Degree of novelty for a surgical technique/device should be mentioned e.g. "first in-human". Not first in human. |  |
|  | **9f** | After the operation, there was no leakage. She started feeding 24 hours after the operation and was discharged after 5 days then she was discharged with a good general condition |  |
| **Follow-up and**  **Outcomes** | **10a** | 6 months of follow up and After the operation, there was no leakage. She started feeding 24 hours after the operation and was discharged after 5 days. | 3 |
|  | **10b** | Important follow-up measures after 6 months of surgery CT was done and was clear of bezoar in stomach. |  |
|  | **10c** | intervention adherence and tolerability was good but the golden role after surgery is to prevent bezoar recurrence |  |
|  | **10d** | Complications and adverse events: upper gastrointestinal endoscopy disclosed a large mass of trichobezoar occupies the stomach which could not be extracted or exceeded. Therefore, the patient underwent emergency laparotomy; an anterior gastrotomy was done (figure 2). There was a giant trichobezoar with the shape of the stomach. We closed the incision in two layers using 2-0 Vicryl suture and the abdomen was closed without drainage |  |
| **Discussion** | **11a** | Trichobezoar diagnosis is made by endoscopic examination and radiography imaging. Upper gastrointestinal (GI) endoscopy can provide information about the structure of the mass. The Computed tomography (CT) investigation can Reveal the existence, localization, and distribution of the bezoars(9).  Different therapeutic modalities have been suggested to treat trichobezoar like endoscopy, Surgery, and pharmacological approaches. Surgery by gastrotomy or enterotomy is still the mainstay for gastric trichobezoar removal especially those that extend into the intestine. Because of the enormous size of the mass, laparotomy was chosen as the surgical method in order to remove the whole trichobezoar mass successfully. | 3 |
|  | **11b** | Bezoars are concretions of foreign substances in the gastrointestinal tract, mainly the stomach. Bezoars composed of hair or hair-like fibers are called 'trichobezoars'. Trichobezoars tend to appear in the second decade of life(3). Often in females with psychiatric disorders including trichotillomania (pulling out their hair) and trichophagia (eating hair).(7)  Trichobezoars form when Ingested hair strands are accumulated in the gastric folds, escaping peristaltic propulsion because of their slippery surface, prevents enough friction which is required to push them out of the stomach. The Ingested hair becomes even more matted together and takes the shape of the stomach, usually as a single solid mass(8). |  |
|  | **11c** | Trichobezoar is caused by chronic ingestion of hair; Small trichobezoars may be extracted by endoscopic fragmentation, huge trichobezoar, on the other hand need surgical removal. Early diagnosis and an appropriate therapy can reduce morbidity and mortality. |  |
|  | **11d** | Early diagnosis and an appropriate therapy can reduce morbidity and mortality.  Psychological counselling plays a pivotal role in order to prevent bezoar recurrence. |  |
| **Patient Perspective** | **12** | We took her perspective on the treatments her received and she was satisfied with the surgery and stated that the condition has not relapsed. | 6 |
| **Informed Consent** | **13** | informed consent was obtained | 6 |
| **Additional Information** | **14** | Ethics and consent to participate: We have the patient’s approval; no more approvals are required. The work has not been published previously.  Consent to publish: Written informed consent was obtained from the patient for publication of this Case Report and any accompanying images. A copy of the written consent is available for review by the Editor of this journal.  Competing interests: Authors declare that there is no conflict of interest.  Availability of data and materials: All data and materials are available. | 6 |
